# Correction: Structure of Type IIL Restriction-Modification Enzyme MmeI in Complex with DNA Has Implications for Engineering New Specificities

**DOI:** 10.1371/journal.pbio.1002471

**Published:** 2016-05-19

**Authors:** The PLOS Biology Staff

## Notice of Republication

This article was republished on April 25, 2016, to correct an error in Figure 5 which was introduced during the typesetting process. The publisher apologises for the error. Please download this article again to view the correct version. The originally published, uncorrected article and the republished, corrected articles are provided here for reference.

## Supporting Information

S1 File Originally published, uncorrected article. (PDF)

S2 File Republished, corrected article. (PDF)
