# Supplementary figures and images for: Structure of Type IIL Restriction-Modification Enzyme MmeI in Complex with DNA Has Implications for Engineering New Specificities
Source: PLoS Biol. 2016 Apr 15;14(4):e1002442. doi: 10.1371/journal.pbio.1002442 (PMC4833311; doi:10.1371/journal.pbio.1002442)

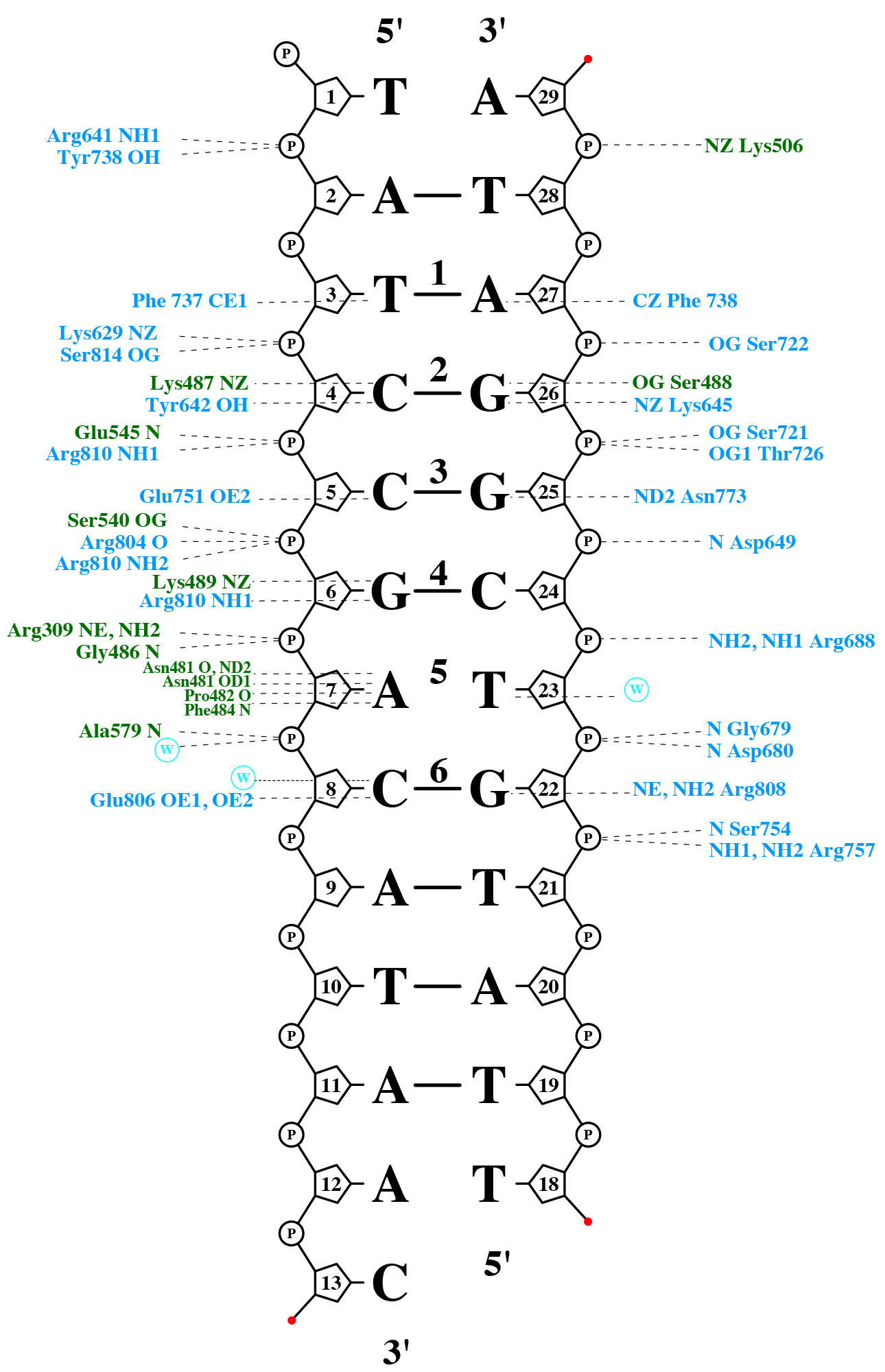

Supplement: S1 Fig — The amino acids dictating specificity of the recognition sequence (labeled 1–6) are depicted directly above the contacting bases. Contacts are only depicted if the distance between bonding atoms is less than 3.5 Å in the crystal structure. (TIF) [file pbio.1002442.s001.tif]

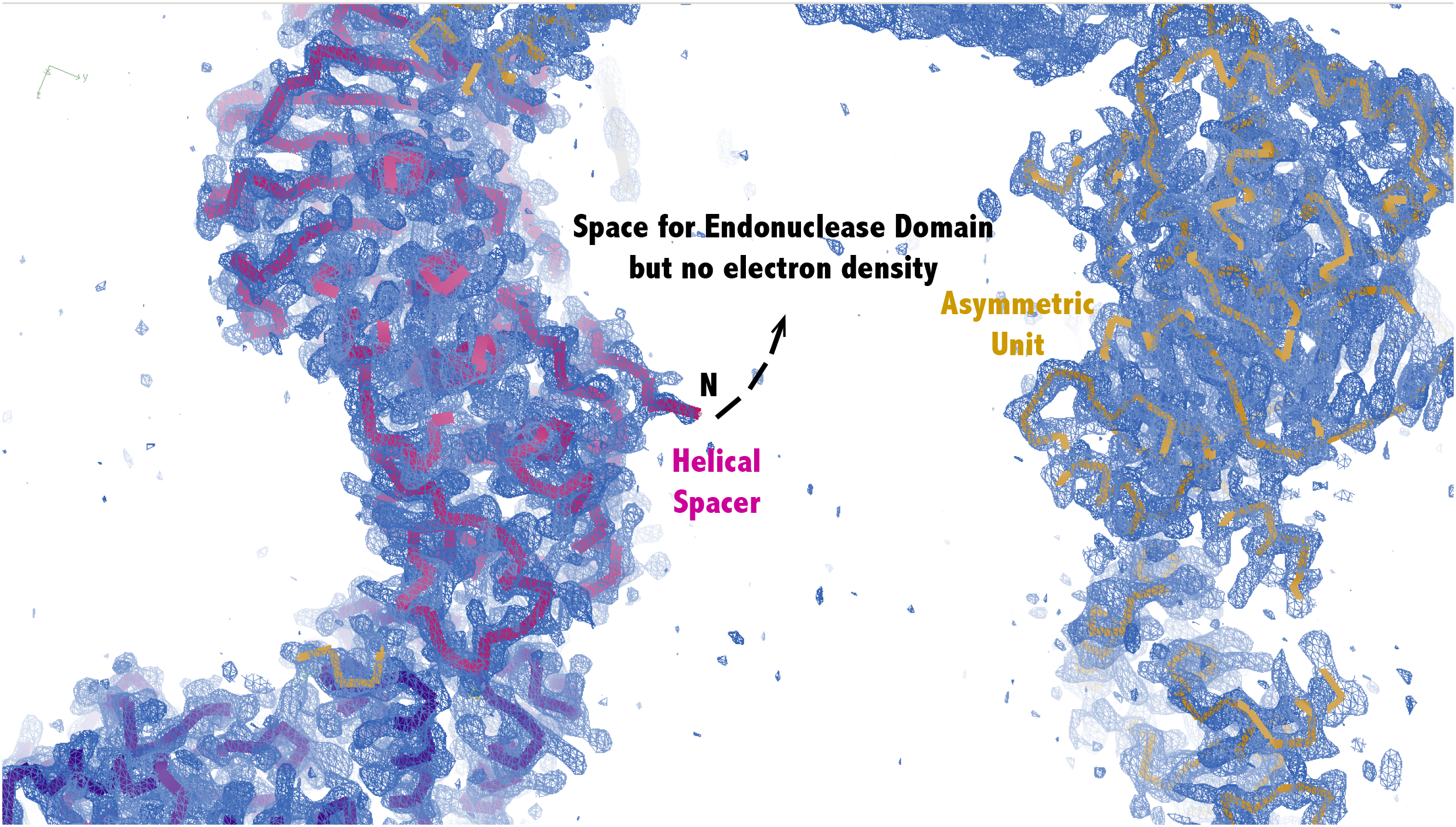

Supplement: S2 Fig — A view of section of a 2Fo-Fc map (contoured 1.3σ) shows absence of electron density for the endonuclease domain, ahead of the helical spacer, suggestive of its disorder or highly mobile nature. (TIF) [file pbio.1002442.s002.tif]
