# Supplementary material for: Structure of Type IIL Restriction-Modification Enzyme MmeI in Complex with DNA Has Implications for Engineering New Specificities
Source: PLoS Biol. 2016 Apr 15;14(4):e1002442. doi: 10.1371/journal.pbio.1002442 (PMC4833311; doi:10.1371/journal.pbio.1002442)
Supplement: S1 Table — (DOCX) [file pbio.1002442.s003.docx]

**S1 Table. MmeI Position 1 mutants**

| Expected Motif | Altered Amino Acids | | | | | Result | Homolog with corresponding amino acid |
| --- | --- | --- | --- | --- | --- | --- | --- |
|  | **Ala723** | | **Phe737** | | **Tyr738** |  |  |
| TCCRAC | Ala | Phe | | Tyr | | Active TCCRAC | MmeI Wildtype |
| ACCRAC | Ala | Arg | | Gln | | Not active | RdeGBII |
| ACCRAC | Ala | Ala | | Gln | | Not active | RlaII, ApyPI |
| CCCRAC | Arg | Gly | | Glu | | Not active | NlaCI |
| CCCRAC | Lys | Asp | | Glu | | Not active | PspPRI |
| GCCRAC | Ser | His | | Val | | Not active | EsaSSI, MchCM4I |
| GCCRAC | Asp | Gln | | Lys | | Not active | NmeAIII, NflHI |
| GCCRAC | Ser | Arg | | Glu | | Not active | AteMI |
| GCCRAC | Asp | Arg | | Glu | | Not active | Awo1030IV |
