# Supplementary material for: Correction: Structure of Type IIL Restriction-Modification Enzyme MmeI in Complex with DNA Has Implications for Engineering New Specificities
Source: PLoS Biol. 2016 May 19;14(5):e1002471. doi: 10.1371/journal.pbio.1002471 (PMC4873199; doi:10.1371/journal.pbio.1002471)
Supplement: S1 File — (PDF) [file pbio.1002471.s001.PDF]

RESEARCH ARTICLE

Structure of Type IIL Restriction-Modification Enzyme MmeI in Complex with DNA Has Implications for Engineering New Specificities

Scott J. Callahan[1], Yvette A. Luyten[2], Yogesh K. Gupta[1], Geoffrey G. Wilson[2], Richard J. Roberts[2], Richard D. Morgan[2]*, Aneel K. Aggarwal[1]*

1 Department of Structural and Chemical Biology, Mount Sinai School of Medicine, New York, New York, United States of America, **2** New England Biolabs Inc., Ipswich, Massachusetts, United States of America

* morgan@neb.com (RDM); aneel.aggarwal@mssm.edu (AKA)

CrossMark
click for updates

OPEN ACCESS

Citation: Callahan SJ, Luyten YA, Gupta YK, Wilson GG, Roberts RJ, Morgan RD, et al. (2016) Structure of Type IIL Restriction-Modification Enzyme MmeI in Complex with DNA Has Implications for Engineering New Specificities. PLoS Biol 14(4): e1002442. doi:10.1371/journal.pbio.1002442

Academic Editor: Gregory A. Petsko, Brandeis University, UNITED STATES

Received: October 21, 2015

Accepted: March 17, 2016

Published: April 15, 2016

Copyright: © 2016 Callahan et al. This is an open access article distributed under the terms of the Creative Commons Attribution License, which permits unrestricted use, distribution, and reproduction in any medium, provided the original author and source are credited.

Data Availability Statement: Atomic coordinates and structure factors are available from the Protein Data Bank. The accession code is 5HR4.

Funding: This work was supported by NIH grant R42-GM105097-01 (RDM and AKA). The funders had no role in study design, data collection and analysis, decision to publish, or preparation of the manuscript.

Competing Interests: YAL, GGW, RJR, and RDM are employees and shareholders of New England Biolabs, Inc.

Abstract

The creation of restriction enzymes with programmable DNA-binding and -cleavage specificities has long been a goal of modern biology. The recently discovered Type IIL MmeI family of restriction-and-modification (RM) enzymes that possess a shared target recognition domain provides a framework for engineering such new specificities. However, a lack of structural information on Type IIL enzymes has limited the repertoire that can be rationally engineered. We report here a crystal structure of MmeI in complex with its DNA substrate and an S-adenosylmethionine analog (Sinefungin). The structure uncovers for the first time the interactions that underlie MmeI-DNA recognition and methylation (5'-TCCRAC-3'; R = purine) and provides a molecular basis for changing specificity at four of the six base pairs of the recognition sequence (5'-T**CCR**A**C**-3'). Surprisingly, the enzyme is resilient to specificity changes at the first position of the recognition sequence (5'-**T**CCRAC-3'). Collectively, the structure provides a basis for engineering further derivatives of MmeI and delineates which base pairs of the recognition sequence are more amenable to alterations than others.

Author Summary

Type II restriction endonucleases (REases) are the bedrock of modern biotechnology. Type II REases were essential for the recombinant DNA revolution and the development of gene technology. However, despite the discovery of more than 4,000 REases, the DNA recognition specificities are limited to only ~365. The recently discovered Type IIL MmeI family of restriction-and-modification (RM) enzymes provides a framework for understanding and engineering new specificities. We report here a crystal structure of MmeI in complex with its DNA substrate and an S-adenosylmethionine analog (Sinefungin). The structure uncovers for the first time the interactions that underlie MmeI-DNA recognition and methylation. The results establish a platform for rationally engineering further

Abbreviations: AdoMet, S-adenosyl methionine; H-bonds, hydrogen bonds; MSA, multiple sequence alignment; MTases, methyltransferases; nts, nucleotides; PET, paired-end tags; REases, restriction endonucleases; RM, restriction-and-modification; r.m.s., root-mean-square; SAD, single-wavelength anomalous diffraction; SAGE, serial analysis of gene expression; TALEN, transcription activator-like effector nuclease; TRD, target recognition domain.

derivatives from MmeI and its homologs that will possess new, intentionally chosen, specificities.

Introduction

Due to their exquisite selectivity, Type II restriction endonucleases (REases) are paradigms in the study of protein-DNA sequence recognition [1,2]. Approximately 4,000 have now been discovered [3], specific for a remarkable 365 different DNA sequences. Impressive as this number is, it represents only a small fraction of the total number of DNA sequences that could in principle be recognized. Attempts to increase the number of REase specificities by protein engineering have met with very limited success due both to our incomplete understanding of the molecular mechanism of recognition and to the proteins themselves, which inherently resist such changes [4–9], a property termed "immutability" [10]. Immutability stems from the circumstances under which these enzymes have evolved. REases occur mainly in prokaryotes—bacteria and archaea—in partnership with DNA-methyltransferases (MTases) of identical specificity that serve to protect the cell's own DNA from REase cleavage [2,11,12]. Together, the two enzymes form a restriction-modification (R-M) system that confers innate immunity against viruses and other infectious genetic elements. Unless compensated for by a corresponding change in the partner enzyme, a change in the specificity of either one is liable to be detrimental due to cleavage of the host's DNA at unprotected sites [10]. Simultaneous, matching changes are exceedingly unlikely among systems in which the REase and MTase(s) are separate proteins that act independently.

Not all R-M systems behave in this way, however. The Type IIG and Type IIL families comprise bifunctional R-and-M (RM) enzymes in which the two catalytic activities share the same target recognition domain (TRD) for sequence recognition [13,14]. These enzymes can change specificity more readily because any change affects both restriction and modification activities in the same way at the same time [15]. There is a selective advantage for cells to switch restriction specificity occasionally to counter resistance among infecting viruses. Accordingly, the TRDs of the bifunctional Type IIL MmeI-family RM enzymes have evolved structures that lend themselves to such changes; as a result, the DNA sequences that these enzymes recognize have diversified very widely [15].

The bifunctional RM enzymes provide a natural platform for engineering new DNA-binding specificities, and some success in this direction has been achieved already [15,16]. The cloning of MmeI, from the bacterium *Methylophilus methylotrophus*, and comparison of its sequence to genome database sequences led to the identification of a family of homologs that, despite significant amino acid similarity, recognize different DNA sequences. Analysis of covariation between the DNA sequences recognized by these enzymes and the amino acid sequences of their TRDs enabled pairs of amino acids specifying several of the base pair positions to be identified [15]. By interchanging these amino acids, derivatives of MmeI and NmeAIII were constructed that recognize new DNA sequences with high fidelity [15]. No structural framework exists for understanding the atomic basis for these specificity changes, however, and this has limited the repertoire that has been rationally engineered in this way.

To better understand the structural basis of DNA recognition and cleavage by Type IIL enzymes, we have determined the crystal structure of MmeI in complex with its DNA substrate. MmeI is a large enzyme (919 amino acids, 105.1 kDa) that integrates DNA recognition and methyltransferase and endonuclease activities within the same polypeptide [13,17,18]. MmeI recognizes the asymmetric DNA sequence 5'-TCCRAC-3' (R = purine; A or G) and

methylates the invariant adenine in the "top" strand (underlined). When multiple unmodified sites are encountered, MmeI cleaves the DNA approximately two helical turns downstream, on average 20 nucleotides (nts) away from the methylated adenine on the top DNA strand and 18 nts away on the bottom DNA strand (thus, TCCRAC 20/18). The structure reveals the amino acids responsible for DNA recognition in MmeI and suggests a basis for the long "reach" of the enzyme between its DNA recognition and cleavage sites. The structure establishes a framework for rationally engineering further derivatives from MmeI and its homologs, which possess new, intentionally chosen specificities.

Results

Structure Determination

MmeI was co-crystallized with a 29-mer DNA duplex containing a single MmeI recognition site (TCCGAC). The co-crystals were obtained in the presence of Sinefungin and diffracted to 2.6 Å resolution with synchrotron radiation. They belong to space group P1 with unit cell dimensions of a = 61.87 Å, b = 95.29 Å, c = 161.96 Å, α = 72.84°, β = 89.15°, and γ = 71.61° (Table 1), and contain two MmeI/DNA/Sinefungin complexes in the crystallographic asymmetric unit. Related by a non-crystallographic symmetry, the two complexes are almost identical (root-mean-square [r.m.s.] deviation ~0.16 Å over 748 Cα). The structure was determined by the single-wavelength anomalous diffraction (SAD) method and refined to 2.6 Å resolution (Table 1). The final refined model consists of two MmeI molecules (residues 156–906), two 13-mer DNA duplexes (sense strand nucleotides 1–13 and antisense strand nucleotides 17–29), two Sinefungin molecules, two calcium ions, and a total of 61 solvent molecules. Regions of protein with no electron density were omitted in model building, and amino acids with weak electron densities for their side chains were modeled as alanines. The current model lacks the endonuclease portion of MmeI due to the lack of electron density for this region.

Overall Architecture

MmeI is composed of five domains. An N-terminal PD-(D/E)XK-type endonuclease domain (residues 1–155) connects to a γ-class *N*6-adenine DNA-methyltransferase domain (6mA-MTase; residues 301–620) via a multi-helical spacer (residues 156–300) (Fig 1A) [19]. These are followed by the TCCRAC-specific TRD (residues 621–825), and a final C-terminal helical bundle (residues 826–919) (Fig 1A). The endonuclease domain is disordered in the present structure, but its putative position—preceding the spacer—is in keeping with the ability of the enzyme to cleave DNA outside of the recognition sequence (Fig 1A). The DNA is embedded between the TRD and the MTase domain with the adenine to be methylated (TCCG**A**C) flipped out of the DNA helix into the catalytic pocket of the MTase domain (Fig 1). The TRD makes contacts to the DNA bases primarily in the major groove, while the MTase domain makes several contacts to the DNA in the minor groove. The primary role of the MTase is to catalyze transfer of the methyl group from S-adenosyl methionine (AdoMet) to the 6-amino group of the target adenine, which resides in the active site cleft of the MTase domain. The overall conformation of the DNA is B-DNA, but it is severely distorted at the juncture where the target adenine is flipped from the helix (Fig 1B). The sugar-phosphate backbone of the target adenine is displaced toward the MTase domain by several Angstroms, and the minor groove over this region widens by ~7.6 Å when compared to a regular B-DNA.

The overall configuration of MmeI can be compared to that of the related Type IIG RM enzyme, BpuSI (878 aa; recognition sequence: GGG**A**C 10/14). BpuSI cleaves roughly one turn of the DNA helix closer to its recognition sequence than MmeI, and creates a 4-base 5'-overhang rather than a 2-base 3'-overhang. The structure of BpuSI has been determined in the

Table 1. Crystallographic parameters and refinement statistics.

	Native MmeI/DNA/Sinefungin	Se-Met MmeI/DNA/Sinefungin
**Space group**	P1	P1
**Cell dimensions**		
**a, b, c (Å)**	61.87, 95.29, 161.96	62.08, 94.68, 159.91
**α, β, γ (°)**	72.84, 89.15, 71.61	73.34, 80.35, 71.89
**Resolution (Å)**	50.0–2.6	50.0–3.0
**R_{sym} (%)[a,b]**	12.8 (51.1)	10.9 (35.9)
**I/σ (I)**	11.6 (2.02)	17.8 (3.0)
**Completeness (%)**	89.3 (75.2)	92.8 (61.8)
**Redundancy**	3.5 (2.9)	3.6 (2.8)
**Phasing Method**		SAD
**Number of heavy atoms sites**		14
**Refinement**		
**Resolution (Å)**	43–2.6	
**No. reflections**	90980	
**R_{factor} (%)[c]/R_{free} (%)[d]**	22.82/25.03	
No. atoms	12,739	
**Protein**	11,578 (residues = 1500)	
**DNA/Sinefungin/Ca^{2+}**	1044/54/2 (50/2/2)	
**Water**	61	
Average B-factors ($Å^2$)		
**Protein**	41.1	
**DNA/Sinefungin**	51/29.7	
**Water/Ca^{2+}**	36.1/38.7	
R.m.s deviations		
**Bond lengths (Å)**	0.01	
**Bond angles (°)**	1.47	
Ramachandran		
**Core region**	97.39	
**Allowed region**	2.47	
**Outliers**	0.13	

[a]Values for outermost shells are given in parentheses.

[b]$R_{sym} = \Sigma| I—<I>|/ \Sigma I$, where I is the integrated intensity of a given intensity.

[c]$R_{factor} = \Sigma|| F_{observed} | -| F_{calculated}|| / \Sigma|F_{observed}|$.

[d]R_{free} was calculated using 2.22% of random data omitted from the refinement of MmeI/DNA/Sinefungin complex.

doi:10.1371/journal.pbio.1002442.t001

absence of DNA and reveals an ordered endonuclease domain that is sequestered by the helical spacer (Fig 2) [14]. Superposition of the MmeI and BpuSI structures suggests that the main conformational change on DNA binding is an ~38° rotation of the TRD to clamp onto the DNA (Fig 2). The MTase domain of MmeI, and to some extent the TRD, also superimpose on M.TaqI (421 aa; recognition sequence: TCG**A**), a monofunctional 6mA-MTase of the same γ-class as MmeI and BpuSI. M.TaqI has been crystallized with and without DNA [20–22]; the position of the bound DNA in the former is nearly identical to that in MmeI. Concomitant with its inability to cleave DNA, M.TaqI lacks the N-terminal cleavage domain of MmeI (and of BpuSI) and the helical connector. It also lacks the C-terminal helical bundle that follows the TRD of MmeI.

DNA Sequence Recognition

The TRD is composed of two α/β subdomains comprising residues 621–745 (TRD-N) and 746–825 (TRD-C). These domains contact the bases of the recognition sequence exclusively in the major DNA groove. TRD-N mainly follows the backbone of the complementary strand of the recognition sequence and interacts with the first two base pairs of the recognition sequence

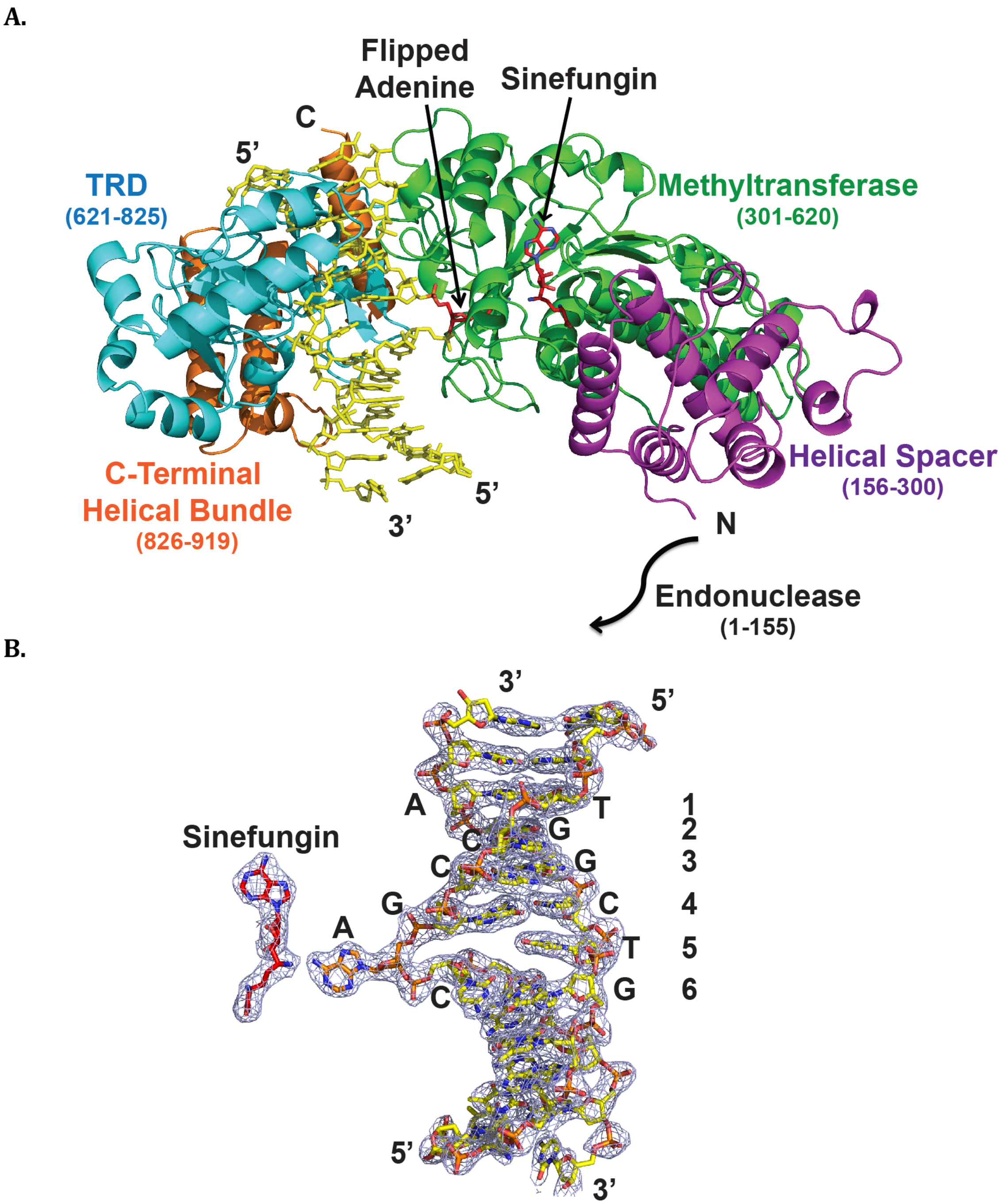

Fig 1. MmeI/DNA/Sinefungin ternary complex. (A) The MmeI helicase spacer (residues 156–300), methyltransferase (residues 301–620), DNA target recognition domain (TRD; residues 621–825), and C-terminal helical region (residues 826–919) are shown in purple, green, cyan, and orange, respectively. DNA is in yellow. The flipped-out adenine (orange) and the bound Sinefungin (red) are labeled. (B) Crystallized DNA and Sinefungin with accompanying 2Fo-Fc map contoured at 1.5 σ. The recognition sequence of TCCGAC is labeled along with the complementary strand.

doi:10.1371/journal.pbio.1002442.g001

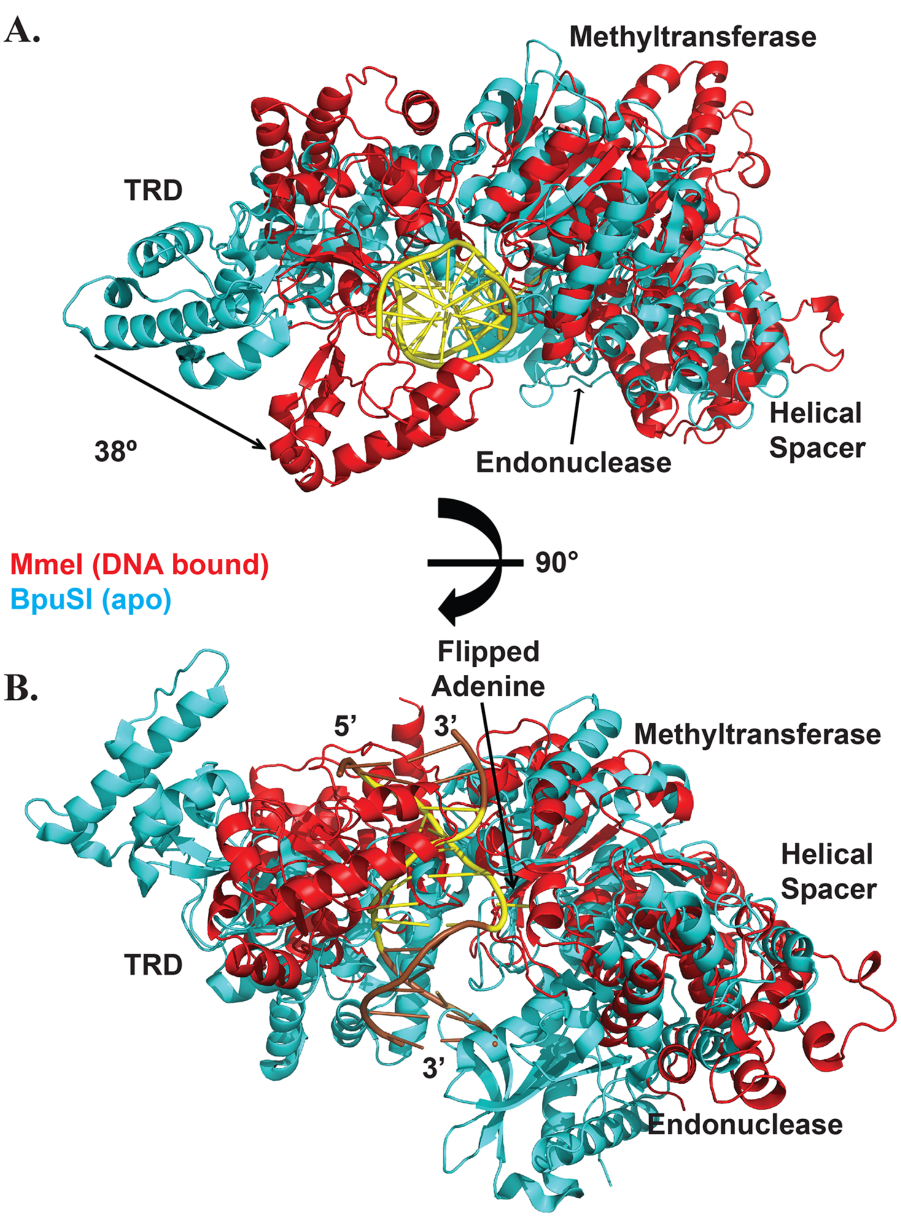

Fig 2. Structural comparison between MmeI and BpuSI. (A) MmeI (red) bound to DNA (yellow) superimposed on apo BpuSI (cyan). The helicase connector, methyltransferase, and DNA target recognition domain (TRD) labels correspond to both structures, while the endonuclease domain is only visible in the BpuSI structure. A comparison of the two structures reveals an ~38° rotation in the TRD, which clamps down on the DNA to make specific contacts. The TRD as a whole shifts by ~27 Å between the two structures. (B) A 90° rotation of the view in (a) to show the relative position of the endonuclease domain.

doi:10.1371/journal.pbio.1002442.g002

(**TC**CGAC). TRD-C tracks the DNA major groove and interacts with the remaining bases (TC**CG**A**C**) (Fig 1A). These interactions are supplemented by contacts in the minor groove from the MTase domain. Altogether, ~2100 Å^2 of solvent-accessible surface area is buried between the DNA and the TRD and the MTase domain (S1 Fig), in the range observed with conventional Type II restriction enzymes such as BamHI and BglII [10,23].

Position 1. The first base pair of the TCCRAC recognition sequence, **T:A**, is specified by three amino acids and appears to be achieved largely without hydrogen bonds (H-bonds).

Tyr738 makes a hydrophobic contact with the thymine 5-methyl group and is positioned by a non-specific H-bond to the phosphate backbone (Fig 3). Phe737 is in van der Waals contact with the thymine *O*4 atom and the *N*6 group of the paired adenine (Fig 3). The Ala723 side chain packs against the N7 and C8 positions of the adenine, in a position where it could presumably exclude a thymine base due to steric clash with the 5-methyl group.

The amino acids at these three positions in MmeI-family enzymes co-vary with the base pair recognized, although not in a simple way. At the Ala723 position, enzymes that recognize C:G have Arg or Lys instead, consistent with canonical contacts between their positively charged side chains and the *O*6/N7 H-bond acceptor atoms of guanine. Enzymes that recognize G:C typically have either a negatively charged carboxyl (Asp or Glu) or a hydroxyl (Ser or Thr) that could H-bond with the cytosine *N*4 donor group. In addition, enzymes that recognize A:T have Ala at position 723, like MmeI, but Gln at position 738 (where MmeI has Tyr), consistent with forming H-bonds at the *N*6/N7 positions of adenine. In contrast, those recognizing C:G typically have Glu at position 738, which could H-bond with cytosine *N*4, and those recognizing G:C typically have a positively charged amino acid at position 737 (MmeI has Phe), which could H-bond with guanine *O*6/N7.

We attempted to rationally alter MmeI specificity at base pair 1 by substituting co-varying amino acids at positions 723, 737, and 738; however, no active mutant enzymes recognizing C:G, G:C, or A:T in place of the wild-type T:A were found (S1 Table). In addition, we replaced the entire loop between Ala723 and Tyr738 with the sequence found in the highly similar enzyme NmeAIII, which recognizes G:C at position 1. This mutant was also found to be inactive. These results indicate that MmeI recognition at position 1 is much less plastic than recognition at the other base pair positions, and that the enzyme is less able to accommodate alternative amino acids within the segment of MmeI TRD apposed to position 1. Overall, it reinforces a notion that residues other than those contacting the bases can also influence specificity [10].

Position 2. The second base pair (**C:G**) appears to be specified predominantly by Tyr642 and Lys645. Tyr642 accepts an H-bond (2.7 Å) from the cytosine *N*4 group, and Lys645 donates bidentate H-bonds to the guanine *O*6 (3.0 Å) and N7 atoms (2.8 Å; Fig 3). In addition, in the minor groove, Lys487 from the MTase domain H-bonds nonspecifically with cytosine *O*2 (2.9 Å), and Ser488 forms two H-bonds with guanine *N*2 (2.9 Å) and N3 (3.3 Å). Thus, all of the hydrogen-bonding atoms and groups of the second base pair are involved in direct H-bonds with MmeI (Fig 3). Among MmeI-family enzymes, lysine at position 645 correlates most frequently with recognition of C:G at position 2, likely due to the bidentate H-bonds to guanine *O*6/N7.

We investigated specificity at position 2 by substituting Tyr642 and Lys645 with residues that correlate with the recognition of alternative base pairs in other family members. A single amino acid change of Lys645 to Met generated an active enzyme with a strong preference for A:T at position 2, and some residual activity towards the wild-type C:G. Purified MmeI K645M enzyme generated a fragment banding pattern consistent with cleavage at TACRAC; however, at the enzyme concentration required for nearly complete cleavage of TACRAC, partial cleavage at TCCRAC was observed as well (Fig 4). Interestingly, on pBR322 DNA, a substrate with 4 TCCRAC sites but no TACRAC sites, little or no cleavage at TCCRAC was observed, suggesting that binding to TCCRAC is substantially less efficient than binding to TACRAC. The double mutant Y642K + K645M changed specificity from C:G to R:Y at position 2 (Fig 4). Thus, altering Tyr642 to Lys in conjunction with the K645M mutation allowed productive binding at G:C in addition to A:T. DNA-methylation data obtained by PacBio SMRT sequencing suggested that this double mutant now has a preference for G:C over A:T (Fig 4). These findings demonstrate that both positions 642 (Tyr) and 645 (Lys) are important for specificity determination at position 2.

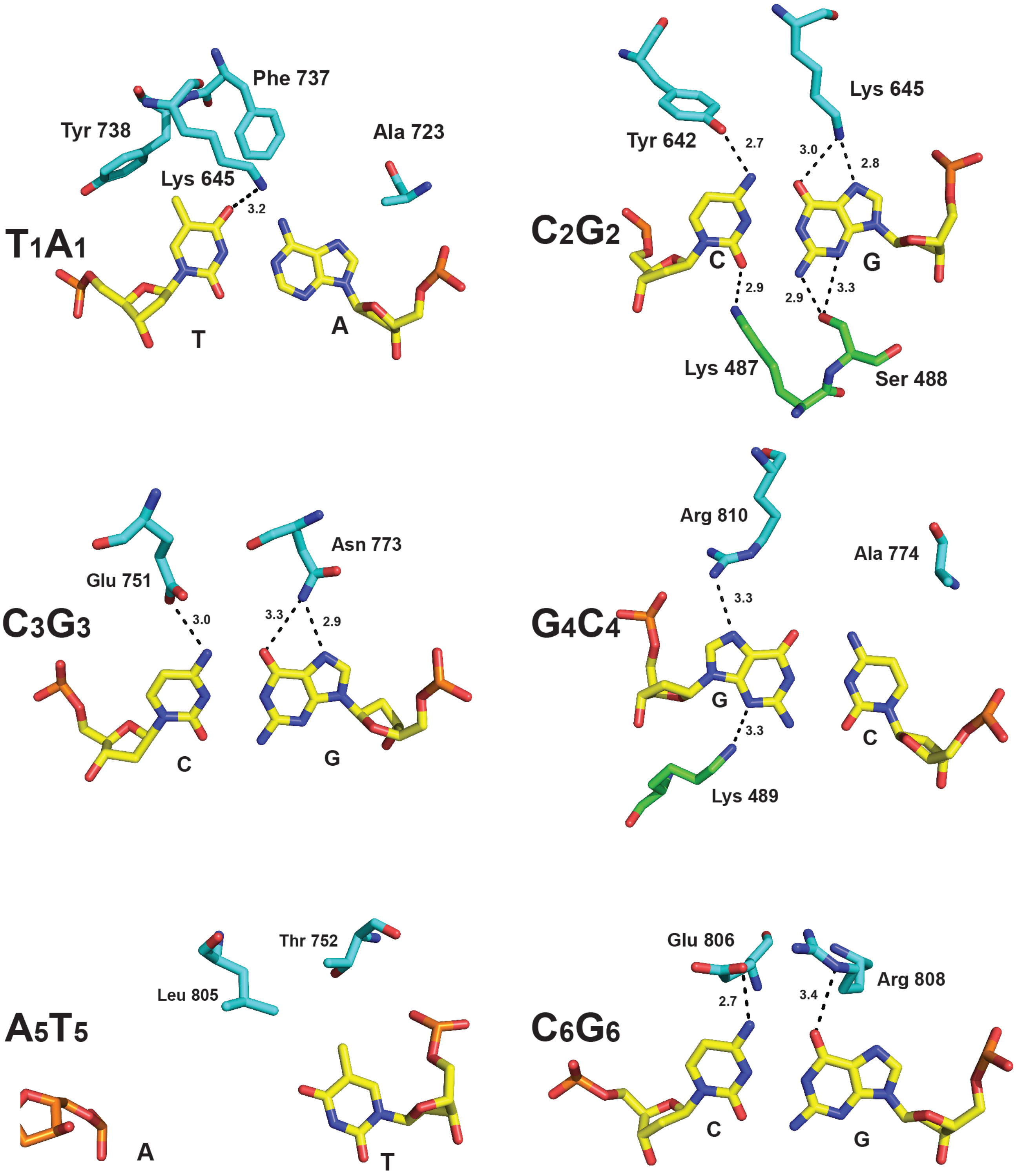

Fig 3. Molecular basis for DNA recognition. The first base pair (T:A) is specified by hydrophobic contacts with Phe737, Tyr 738, and Ala723 of the TRD; the second base pair (C:G) makes contacts with Lys645 and Tyr642 of the TRD and Lys487 and Ser488 of the MTase domain; the third base pair (C:G) is specified by Glu751 and Asn773 of the TRD; the fourth base pair (R:Y) makes contacts with Arg810 of the TRD and Lys489 of the MTase domain; at the fifth base pair (A:T), the thymine opposite the adenine to be methylated is specified by hydrophobic contacts with Thr752 and Leu805 of the TRD; the sixth base pair (C:G) is specified by Glu806 and Arg808 of the TRD. H-bonds are depicted by dashed lines and distances in Angstroms.

doi:10.1371/journal.pbio.1002442.g003

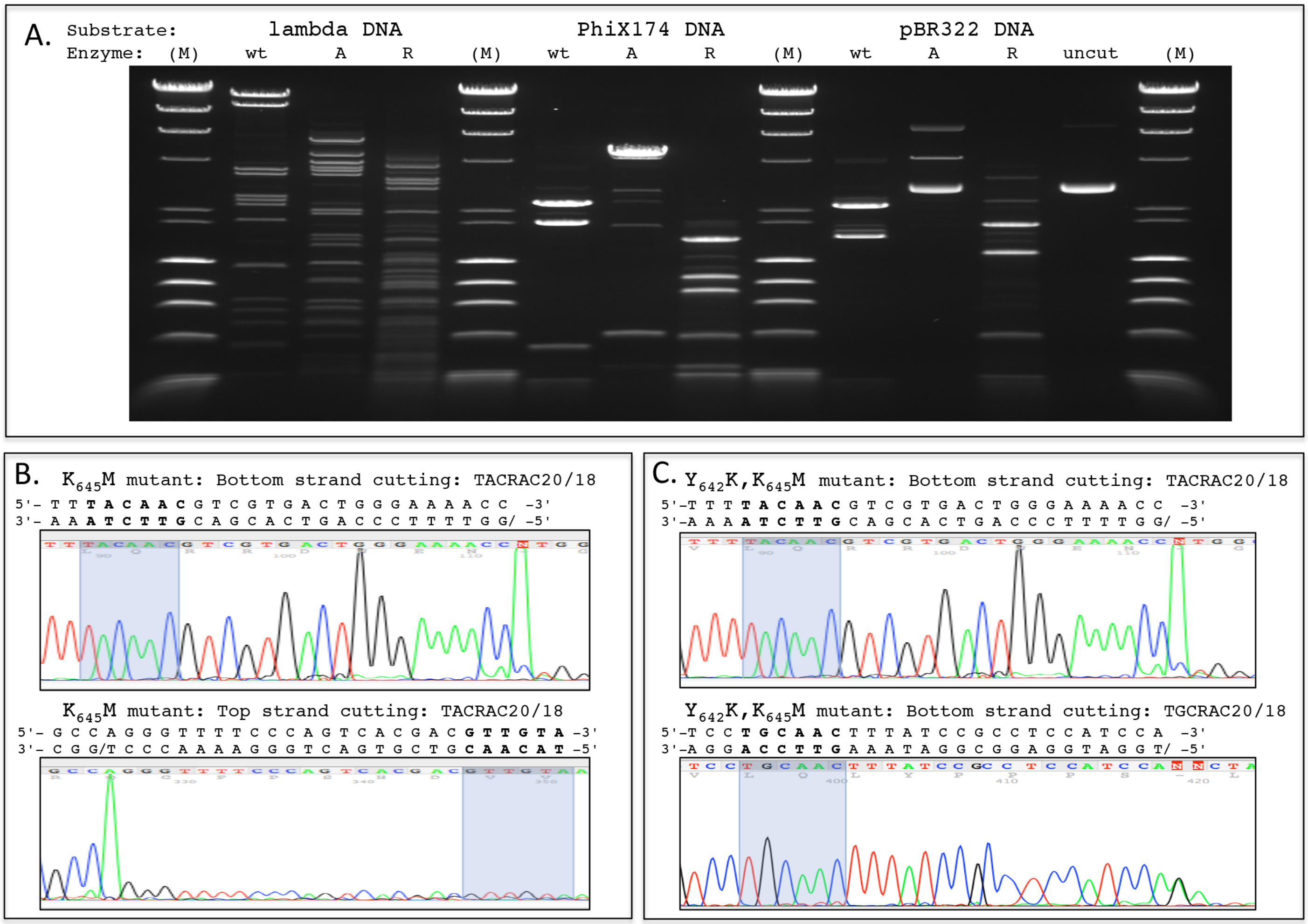

Fig 4. Change in specificity at position 2. (A) Restriction fragment digestion patterns of lambda, PhiX174, and pBR322 DNAs with wt = wild type MmeI, which cuts at TCCRAC20/18; A = MmeI Lys_{645}Met mutant, which cuts at TACRAC20/18; R = MmeI Tyr_{642}Lys, Lys_{645}Met double mutant, which cuts at TRCRAC20/18; M = size standard, lambda-HindIII digest plus PhiX174-HaeIII digest. (B) Cut site determination for MmeI K_{645}M mutant showing cutting at TACRAC20/18. Run-off Sanger sequencing of pUC19 DNA (TACRAC site at 376 to 381), priming from both sides (5' and 3') of the TACRAC recognition site and point of DNA cleavage. (C) Cut site determination for MmeI Y_{642}K, K_{645}M double mutant showing cutting at both TACRAC20/18 (top panel, pUC19 site at 376 to 381) and TGCRAC (bottom panel, pUC19 site at 1842 to 1847). Run-off Sanger sequencing of the cleaved pUC19 DNA, showing priming from 5' to the TACRAC or TGCRAC recognition site (bottom strand cleavage shown).

doi:10.1371/journal.pbio.1002442.g004

The amino acids specifying the third, fourth, and sixth base pairs (TC**C**R**A**C) confirm our predictions from earlier multiple sequence alignments (MSAs) and covariation analyses [15]. Glu751 and Asn773 specify position 3 (**C**:G), Arg810 and Ala 774 specify the ambiguous position 4 (**R**:Y), and Glu806 and Arg808 specify position 6 (**C**:G; Fig 3).

Position 3. At base pair 3 (TC**C**GAC), Glu751 forms an H-bond with cytosine *N*4 (3.0 Å), and the amido nitrogen (ND2) of Asn773 forms bidentate H-bonds with guanine *O*6 (3.3 Å) and N7 (2.9 Å). We have shown previously that substitution of Glu751 by lysine or arginine, and of Asn773 by aspartate (D), changes the specificity of the enzyme from C:G to G:C at this position [15], mimicking the amino acid combinations that occur naturally in several MmeI-family enzymes with this specificity.

Position 4. At base pair 4 (TCC**G**AC), Arg810 forms a single H-bond with guanine N7 (3.3 Å). In the minor groove, Lys489 forms an H-bond with the guanine N3 atom (3.3 Å), but since all four bases have an H-bond acceptor at this location, this H-bond is nonspecific. The

H-bond between Arg810 and guanine N7 could form equally well with adenine, and so the conformation of Arg810 is consistent with the ability of MmeI to recognize either purine base, G or A (i.e., R), at this position (Fig 3). Nonetheless, many MmeI-family enzymes are specific for just G:C at this position. Specificity for G:C rather than R:Y appears to correlate with the presence of a bulky amino acid at position 774 instead of the alanine in MmeI. We propose that the bulky amino acid obstructs thymine by sterically clashing with the thymine 5-methyl group, thereby preventing an A:T base pair from occupying position 4. Indeed, in our previous study, when we replaced Ala774 in MmeI with leucine, the A774L mutant recognized only a G:C at base pair 4 [15]. In addition, we found that whereas wild-type MmeI recognized and cleaved modified sequences containing 5-methylcytosine (5mC) at this position, the A774L mutant could no longer cleave the 5mC-modified sequence, consistent with the importance of the residue at position 774 in specifying R:Y or G:C at this position [15].

Position 5. At base pair five (TCCG**A**C), the thymine opposite the adenine to be methylated is specified by hydrophobic contacts with Thr752 and Leu805 (Fig 3), both well conserved among MmeI family members. The adenine itself is flipped out of the DNA helix and enters the catalytic cleft of the MTase domain. Interestingly, the guanine at position 4 (TCC**G**AC) is highly buckled, and its sugar moiety partially occupies the space vacated by the target adenine (Fig 1B). Thus, the configuration of base pair 4 might contribute to the flipping of target adenine from the DNA helix.

Position 6. At base pair 6 (TCCGA**C**), the side chains of Glu806 and Arg808 are fixed in position by a salt link. Glu806 forms one H-bond with cytosine *N*4, and Arg808 forms one H-bond with guanine *O*6. Most MmeI family enzymes recognize either C:G or G:C at base pair 6, and the identities of amino acids at positions 806 and 808 correlate closely with specificity. The Glu806 and Arg808 pair (E-R) exclusively specifies **C**:G, and the Lys806-Asp808 pair (K-D) exclusively specifies **G**:C. Accordingly, in our previous study, when we changed the E-R pair in MmeI to K-D, the mutant switched specificity from TCCRA**C** to TCCRA**G** [15]. We suspect that the K-D pair is also stabilized by a salt bridge and makes analogous H-bonds with a G:C base pair at this position.

DNA Methylation

The MTase domain (aa ~301–620) consists of a twisted β-sheet flanked by α-helices on both sides (Fig 1A). The two principal motifs characteristic of amino-methyltransferases, generically termed "FGG" (motif I = AdoMet-binding site, aa 360–370) and "DPPY" (motif IV = nucleotide binding and catalytic site, aa 481–484) extend from adjacent loops that connect secondary structure elements. Based on the order and sequences of these motifs, MmeI belongs to the γ class of amino-methyltransferases [19], in which motif I is typically . . .FDPACGCGXFL. . . and motif IV, . . .NPPF. . .. The extrahelical adenine (TCCR**A**C) occupies the catalytic cleft between motifs I and IV and forms three H-bonds with residues of the catalytic-site. Consistent with other γ-class (but not with *β*-class) amino-methyltransferases [24], motif IV residues face the Hoogsteen-edge of the flipped adenine base. The adenine N7 atom accepts one H-bond from the Phe484 main chain N (2.6 Å), and the *N*6-group donates one H-bond to Asn481 OD1 (2.8 Å) and one to Pro482 main chain O (2.7 Å) (Fig 5). A fourth, weak, H-bond might also be present between adenine N1 and Asn481 ND2 (3.5 Å). The extrahelical adenine is further stabilized by π–π interactions with the aromatic rings of His314, Phe484, and Trp570, which form a box around the base. His314 stacks on one side of the adenine, Phe484 stacks on the other, and Trp570 stacks edge-on (Fig 5). All of these amino acids (belonging to the NPPF motif IV, as well as His314 and Trp570) are absolutely conserved in the 341 MmeI-family enzymes whose sequences we have aligned to date.

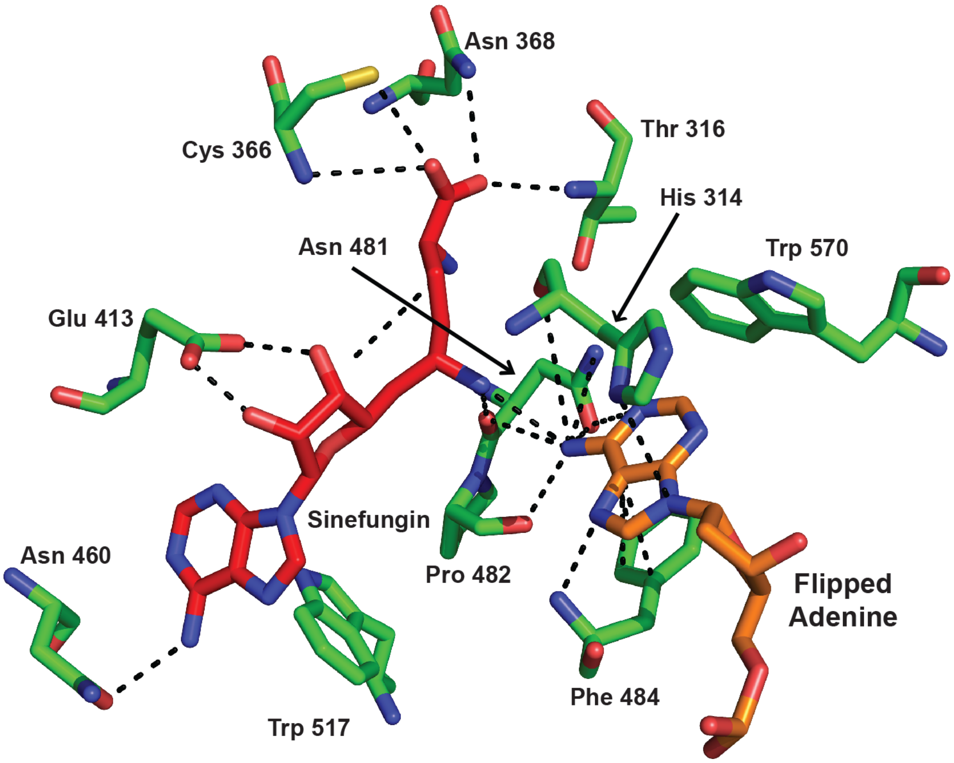

Fig 5. Interactions with Sinefungin. The SAM analog Sinefungin is tightly bound within the MTase domain via extensive hydrogen bonding (dashed lines) and hydrophobic contacts.

doi:10.1371/journal.pbio.1002442.g005

The acceptor atoms of Asn481 and Pro482 to which adenine *N*6 donates H-bonds lie above the plane of the flipped base, suggesting that the nitrogen atom possesses a tetrahedral, SP3, orbital geometry, rather than the planar SP2 geometry it possesses when intrahelical. In this induced SP3 configuration, the electronegative lone pair orbital of the nitrogen points directly toward the electropositive methyl group of AdoMet modeled into our structure, appropriately positioned for methyl transfer by in-line nucleophilic attack (Fig 5). To avoid catalysis and methyl transfer in our complexes, we crystallized MmeI in the presence of the AdoMet analog, Sinefungin, which has a nontransferable amino group in place of the methyl group. This amino group is positioned 3.4 Å from the adenine *N*6 atom in our structure and is slightly displaced. When we aligned the structure of MmeI with that of M.TaqI (pdb:2ADM), which was crystallized with AdoMet [21,22], the cofactor and analog superimposed closely, and the methyl group of AdoMet was found to be closer to the adenine *N*6 atom (3.0 Å) and in slightly better alignment.

DNA Cleavage

MmeI-family enzymes have the longest "reach" among Type II REases, cleaving DNA with some variability 21-22-nt away from the methylated adenine in the "top" DNA strand, and 19-20-nt away in the complementary, "bottom," strand. In the majority of these enzymes, the methylated A is the penultimate base in the recognition sequence, and so most cleave approximately 20/18 downstream from the sequence. By comparison, FokI, a Type IIS REase in which the DNA recognition and cleavage functions are also located on separate domains, cleaves DNA 9-nt/13-nt downstream of the recognition sequence [25]. The ability of MmeI to generate 20-bp "tags" has made it an attractive enzyme for certain applications, including serial analysis

of gene expression (SAGE) and paired-end tags (PET) in next-generation DNA sequencing. Although the cleavage domain of MmeI (residues 1–155) cannot be seen in our structure (S2 Fig), its putative position, far from the TRD, is consistent with the ability of MmeI to cleave some distance away from the recognition sequence (Fig 1A). The helical spacer likely plays a key role in positioning the cleavage domain correctly in this regard, 20-nt/18-nt, from the sequence recognized.

Amino acid sequence analysis of MmeI family enzymes indicates that each contains only one catalytic site, belonging to the PD. . .(D/E)XK nuclease superfamily [11,12]. The two parts of this motif, PD and (D/E)XK, usually form the termini of adjacent β-strands and fold such that the acidic residues (D and E) coordinate one or more divalent metal ions, and the lysine (K) contributes to activation of a hydrolytic water molecule [11,12]. In the case of MmeI, the catalytic residues are V69-D70. . .E80-M81-K82, and mutation of D70, E80, or K82 to alanine eliminates endonuclease activity [26]. REases generally cleave both strands of duplex DNA in one binding event, and so their active forms are often multimeric, comprising two, four, and sometimes more identical subunits [12,27]. At a minimum, MmeI must cleave DNA as a dimer in which the catalytic domains of two molecules interact and each cleave one DNA strand. There is "vacant" space in the crystals adjacent to the helical spacer that can accommodate a domain of the size of the cleavage domain. The lack of electron density in this region (S2 Fig) suggests that the cleavage domain is mobile and flexibly tethered to the helical spacer, and that it may only become ordered when dimerized with that of a second enzyme molecule to form a competent cleavage complex. A similar pattern (disordered endonuclease domain in the crystal) was also observed in structures of a Type III RM enzyme EcoP15I [24] and a Type IIS enzyme AspBHI [28]. Unlike Type IIG BpuSI, MmeI requires two DNA recognition sites for efficient DNA cleavage, suggesting that both molecules must be bound to recognition sites in order to dimerize productively.

Discussion

We present here the first crystal structure of a Type IIL RM enzyme bound to its DNA substrate. MmeI differs from conventional Type II R-M systems (such as BamHI or EcoRI) in that the DNA recognition, methyltransferase, and endonuclease activities reside within the same polypeptide. The fact that the same DNA recognition module is responsible for host modification and endonuclease functions makes MmeI (and related enzymes) much more amenable to changes in DNA-binding and -cleavage specificities than conventional Type II enzymes. Based on bioinformatics analysis alone, we have rationally engineered dozens of MmeI-like enzymes with new specificities [15]. These specificity changes are at positions 3, 4, and 6 of the MmeI recognition sequence (TC**CR**A**C**), and the engineered enzymes have specific activities that are comparable to the wild-type enzyme. The DNA-bound MmeI structure provides a molecular basis for these specificity changes and reveals new interactions to guide the engineering of additional enzymes.

Overall, MmeI recognizes base pairs 3, 4, and 6 (TC**CR**A**C**) in a similar manner to that anticipated from previous bioinformatics analyses. As anticipated, base pair 3 is recognized by Glu751 and Asn773; base pair 4 by Arg810 and Ala774; and base pair 6 by Glu806 and Arg808. This convergence between structure and bioinformatics analysis shows the utility of covariation analyses using MSAs in predicting amino acids that recognize DNA in Type IIL REases. The structure provides atomic-level details on how Glu751, Asn773, Arg810, Ala774, Glu806, and Arg808 actually interact with DNA and a basis for specificity changes reported previously, including C:G to G:C at position 3, R:Y to G:C at position 4, and C:G to G:C at position 6 (Fig 6).

Notably, previous bioinformatics and MSA covariation analyses did not yield insights into how MmeI (and related enzymes) recognizes DNA at positions 1 and 2. Our structure suggests

that the T:A base pair at position 1 (**T**CCRAC) is specified mainly by hydrophobic interactions between Tyr738 and the 5-methyl group of T. Interactions with the C:G base pair at position 2 are more extensive than to other base pairs, with specific hydrogen bond contacts from the major (Tyr642 and Lys645) and minor (Lys487/Ser488) groove sides. Previous sequence covariation analyses failed to pinpoint the positions corresponding to MmeI Tyr642 and Lys645 as specifying recognition at position 2, because similar amino acid residues at these positions give rise to different sequence specificities in various MmeI family enzymes. For example, isoleucine and lysine at these positions, respectively, results in recognition of an A:T base pair in EsaSSI, MchCM4I, and AquIII, but C:G base pair in RmuAI. Several other enzymes that recognize an A:T base pair at this position contain a methionine at the position corresponding to Lys645, paired with either tyrosine (NlaCI) or phenylalanine (SdeAI, CstMI) at the position corresponding to Tyr642. Accordingly, when we change Lys645 to methionine in MmeI, the altered enzyme now preferentially recognizes an A:T base pair at position 2 (Fig 4), though it retains some partial activity toward the wild-type C:G base pair. It is likely that hydrophobic interactions between the methionine and the 5-methyl group of T contribute to this preference (Fig 6). Tyr642 seems readily able to contact an adenine in place of a cytosine, likely making similar interactions with the adenine *N*6 as with the cytosine *N*4 (Fig 6). Changing Tyr642 to Lys in combination with Lys645Met resulted in recognition of R (both A:T and G:C) at position 2. MmeI homologs that recognize a G:C base pair at position 2 also have Lys or Arg at the 642 position (RflFIII), often paired with Gln at position 645. In our modeling, Lys642 appears well positioned to contact the N7 of the purine (A or G) and may be localized for this contact by interaction with the backbone carbonyl of Asn773 and the hydroxyl of Tyr776. These results demonstrate the importance of both Tyr642 and Lys645 positions in specifying recognition at position 2 in the MmeI family enzymes.

Overall, the creation of enzymes with programmable DNA-binding and -cleavage specificities has been a goal ever since the discovery of REases more than 40 y ago. However, attempts to rationally alter the DNA recognition specificities of conventional Type II REases have met with very limited success. Most of the current effort has thus shifted to artificial nucleases such as Zinc Finger Nucleases and transcription activator-like effector nucleases (TALENs), or to homing endonucleases [29–36]. The many recently discovered MmeI-like enzymes offer an alternative approach to achieve "true" REase specificity engineering. The fact that a single DNA recognition module is responsible for host modification and restriction in these enzymes allows for rapid evolution of new specificities. The MmeI structure provides a basis for beginning to understand how Type IIL enzymes like MmeI recognize their DNA substrates and a framework for changing their specificities.

Materials and Methods

Protein Expression and Purification

Both the native and selenium-methionine (Se-met) MmeI proteins were expressed and purified as described previously [37].

Crystallization and Structure Determination

The native MmeI protein was successfully crystalized in complex with a 29-mer DNA using 2 μl hanging-drops over 1 ml reservoirs at 293 K. The optimized crystals were grown using a mother liquor of 20% PEG 4K, 0.1M Hepes (pH7.5), and 0.1M $(NH_4)_2SO_4$. Resolution was improved to 2.6 Å by replacing several thymines outside of the recognition site with 5-bromouracil (5'TATCCGACAUAACGCUAGUCACUAGCUUC-3'/3'ATAGGCTGUATUGCGAU CAGUGAUCGAAG-5'; where U is 5-bromouracil). The brominated DNA oligonucleotides

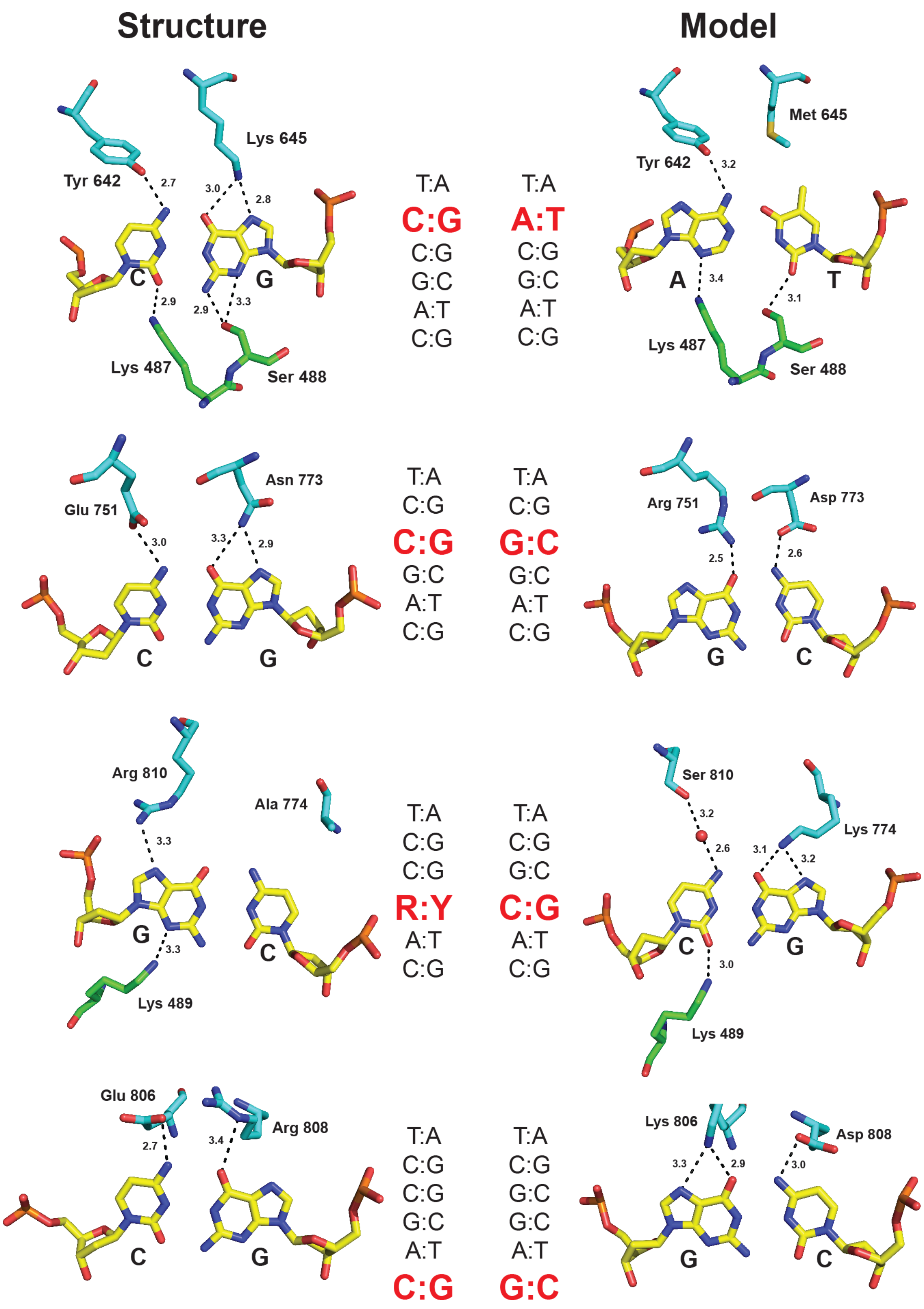

Fig 6. Molecular basis for DNA specificity changes at position 2, 3, 4, and 6. Contacts in the structure are shown on the left and the specificity changes are modeled on the right. At position 2, mutation of Lys645 to Met645 converts DNA specificity from C:G to A:T; at position 3, mutation of Glu751 and Asn773 to Arg751 and Asp773 converts DNA specificity from C:G to G:C; at position 4, mutation of Arg810 and Ala774 to Ser810 and Lys774 converts DNA specificity from G:C to C:G; at position 6, mutation of Glu806 and Arg808 to Lys806 and Asp808 converts DNA specificity from C:G to G:C.

doi:10.1371/journal.pbio.1002442.g006

were synthesized at New England Biolabs and PAGE purified prior to crystallization. For cryo-protection, the crystals were soaked for 5 min in solutions containing mother liquor plus increasing concentrations of glycerol (final concentration of 30% glycerol) and plunged into liquid nitrogen. Given the absence of an appropriate molecular replacement solution, co-crystals with Se-met MmeI (14 methionines per molecule) were grown under similar conditions as the native enzyme. The Se-met crystals diffracted to 3.0 Å resolution.

The X-ray diffraction data on the MmeI/DNA/Sinefungin co-crystals were measured at the Advanced Photon Source at the Argonne National Laboratory. The data on native crystals were measured at beamline 23ID-D at a wavelength of 0.91938 Å, while single wavelength anomalous data on a Se-Met crystal were measured at a wavelength of 0.97944 Å (Se-K absorption edge) at the beamline 24ID-C. The HKL2000 package [38] was used to merge and scale X-ray data. Both the native and Se-Met crystals belong to space group P1. The unit-cell dimensions of native crystals are a = 61.87 Å, b = 95.29 Å, c = 161.96 Å, α = 72.84°, β = 89.15°, and γ = 71.61°; and unit-cell dimensions of the Se-Met crystals are a = 62.08 Å, b = 94.68 Å, c = 159.91 Å, α = 73.34°, β = 80.35°, and γ = 71.89°. The structure was solved using SAD phasing method using SHARP [39]. The electron density map derived from experimental phasing was readily interpretable and showed clear electron density of both protein and DNA molecules. The model was built manually using program Coot [40] and iteratively refined with the program package Phenix [41] to the 2.6 Å resolution limit of the native crystals (Table 1). The final model contains two molecules of MmeI bound to two separate DNA duplexes and two Sinefungin moieties. The quality of the structure is excellent, with >97% of the residues in the most favored regions of the Ramachandran plot (Table 1).

DNA Cleavage Assay

Endonuclease activity was assayed by incubating various amounts of MmeI (wt or mutant) enzyme for 30 min at 37°C in NEBuffer 4 (20 mM Tris-acetate, pH 7.9, 10 mM magnesium acetate, 50 mM potassium acetate, 1 mM DTT) supplemented with AdoMet at 80 μM, containing 1 μg substrate DNA per 50 μl. Reactions were terminated by the addition of loading dye (NEB B7024) and reaction products were analyzed by gel electrophoresis in 1% LE agarose gels.

Supporting Information

S1 Fig. A schematic of amino acid–nucleic acid contacts in the crystal structure. The amino acids dictating specificity of the recognition sequence (labeled 1–6) are depicted directly above the contacting bases. Contacts are only depicted if the distance between bonding atoms is less than 3.5 Å in the crystal structure.
(TIF)

S2 Fig. Electron density map. A view of section of a 2Fo-Fc map (contoured 1.3σ) shows absence of electron density for the endonuclease domain, ahead of the helical spacer, suggestive of its disorder or highly mobile nature.
(TIF)

S1 Table. MmeI Position 1 mutants.
(DOCX)

Acknowledgments

We thank the staff at beamlines 24ID and 23ID at the Advanced Photon Source (APS) and beamline X29A at Brookhaven National Laboratory (BNL) for facilitating data collection. We

thank K. Rajashankar (beamline 24ID) for useful suggestions and R. Jain for help during early stages of the project and for useful suggestions.

Author Contributions

Conceived and designed the experiments: RJR GGW RDM AKA. Performed the experiments: SJC YAL RDM. Analyzed the data: SJC YAL YKG GGW RJR RDM AKA. Wrote the paper: SJC YKG GGW RJR RDM AKA.

References

1. Pingoud A, Wilson GG, Wende W. Type II restriction endonucleases—a historical perspective and more. Nucleic acids research. 2014; 42(12):7489–527. doi: 10.1093/nar/gku447 PMID: 24878924; PubMed Central PMCID: PMCPMC4081073.
2. Roberts RaH SE. Type II restriction endonucleases. In: Linn SM L, RS, and Roberts RJ, editor. Nucleases. New York: Cold Spring Harbor; 1993. p. 35–88.
3. Roberts RJ, Vincze T, Posfai J, Macelis D. REBASE—a database for DNA restriction and modification: enzymes, genes and genomes. Nucleic acids research. 2015; 43(Database issue):D298–9. doi: 10.1093/nar/gku1046 PMID: 25378308; PubMed Central PMCID: PMCPMC4383893.
4. Jeltsch A, Wenz C, Wende W, Selent U, Pingoud A. Engineering novel restriction endonucleases: principles and applications. Trends in biotechnology. 1996; 14(7):235–8. doi: 10.1016/0167-7799(96)10030-5 PMID: 8771796.
5. Lanio T, Jeltsch A, Pingoud A. On the possibilities and limitations of rational protein design to expand the specificity of restriction enzymes: a case study employing EcoRV as the target. Protein engineering. 2000; 13(4):275–81. PMID: 10810159.
6. Dorner LF, Bitinaite J, Whitaker RD, Schildkraut I. Genetic analysis of the base-specific contacts of BamHI restriction endonuclease. J Mol Biol. 1999; 285(4):1515–23. doi: 10.1006/jmbi.1998.2408 PMID: 9917393.
7. Heitman J, Model P. Mutants of the EcoRI endonuclease with promiscuous substrate specificity implicate residues involved in substrate recognition. EMBO J. 1990; 9(10):3369–78. PMID: 2209548; PubMed Central PMCID: PMCPMC552075.
8. Samuelson JC, Xu SY. Directed evolution of restriction endonuclease BstYI to achieve increased substrate specificity. J Mol Biol. 2002; 319(3):673–83. doi: 10.1016/S0022-2836(02)00343-1 PMID: 12054862.
9. Osuna J, Flores H, Soberon X. Combinatorial mutagenesis of three major groove-contacting residues of EcoRI: single and double amino acid replacements retaining methyltransferase-sensitive activities. Gene. 1991; 106(1):7–12. PMID: 1937043.
10. Lukacs CM, Kucera R, Schildkraut I, Aggarwal AK. Understanding the immutability of restriction enzymes: crystal structure of BglII and its DNA substrate at 1.5 A resolution [see comments]. Nat Struct Biol. 2000; 7(2):134–40. PMID: 10655616
11. Aggarwal AK. Structure and function of restriction endonucleases. Curr Opin Struct Biol. 1995; 5:11–9. PMID: 7773740
12. Pingoud A, Fuxreiter M, Pingoud V, Wende W. Type II restriction endonucleases: structure and mechanism. Cell Mol Life Sci. 2005; 62(6):685–707. PMID: 15770420.
13. Morgan RD, Bhatia TK, Lovasco L, Davis TB. MmeI: a minimal Type II restriction-modification system that only modifies one DNA strand for host protection. Nucleic acids research. 2008; 36(20):6558–70. Epub 2008/10/22. gkn711 [pii] doi: 10.1093/nar/gkn711 PMID: 18931376; PubMed Central PMCID: PMC2582602.
14. Shen BW, Xu D, Chan SH, Zheng Y, Zhu Z, Xu SY, et al. Characterization and crystal structure of the type IIG restriction endonuclease RM.BpuSI. Nucleic acids research. 2011; 39(18):8223–36. Epub 2011/07/05. gkr543 [pii] doi: 10.1093/nar/gkr543 PMID: 21724614; PubMed Central PMCID: PMC3185434.
15. Morgan RD, Luyten YA. Rational engineering of type II restriction endonuclease DNA binding and cleavage specificity. Nucleic acids research. 2009; 37(15):5222–33. Epub 2009/07/02. gkp535 [pii] doi: 10.1093/nar/gkp535 PMID: 19567736; PubMed Central PMCID: PMC2731914.
16. Morgan RD, Dwinell EA, Bhatia TK, Lang EM, Luyten YA. The MmeI family: type II restriction-modification enzymes that employ single-strand modification for host protection. Nucleic acids research. 2009; 37(15):5208–21. Epub 2009/07/07. gkp534 [pii] doi: 10.1093/nar/gkp534 PMID: 19578066; PubMed Central PMCID: PMC2731913.

17. Boyd AC, Charles IG, Keyte JW, Brammar WJ. Isolation and computer-aided characterization of MmeI, a type II restriction endonuclease from Methylophilus methylotrophus. Nucleic acids research. 1986; 14 (13):5255–74. Epub 1986/07/11. PMID: 3016643; PubMed Central PMCID: PMC311539.
18. Tucholski J, Zmijewski JW, Podhajska AJ. Two intertwined methylation activities of the MmeI restriction-modification class-IIS system from Methylophilus methylotrophus. Gene. 1998; 223(1–2):293–302. Epub 1998/12/22. PMID: 9858752.
19. Malone T, Blumenthal RM, Cheng X. Structure-guided analysis reveals nine sequence motifs conserved among DNA amino-methyltransferases, and suggests a catalytic mechanism for these enzymes. J Mol Biol. 1995; 253(4):618–32. Epub 1995/11/03. S0022-2836(85)70577-3 [pii] doi: 10.1006/jmbi.1995.0577 PMID: 7473738.
20. Goedecke K, Pignot M, Goody RS, Scheidig AJ, Weinhold E. Structure of the N6-adenine DNA methyltransferase M.TaqI in complex with DNA and a cofactor analog. Nat Struct Biol. 2001; 8(2):121–5. PMID: 11175899.
21. Labahn J, Granzin J, Schluckebier G, Robinson DP, Jack WE, Schildkraut I, et al. Three-dimensional structure of the adenine-specific DNA methyltransferase M.Taq I in complex with the cofactor S-adenosylmethionine. Proc Natl Acad Sci U S A. 1994; 91(23):10957–61. Epub 1994/11/08. PMID: 7971991; PubMed Central PMCID: PMC45145.
22. Schluckebier G, Kozak M, Bleimling N, Weinhold E, Saenger W. Differential binding of S-adenosylmethionine S-adenosylhomocysteine and Sinefungin to the adenine-specific DNA methyltransferase M.TaqI. J Mol Biol. 1997; 265(1):56–67. Epub 1997/01/10. S0022-2836(96)90711-1 [pii] doi: 10.1006/jmbi.1996.0711 PMID: 8995524.
23. Newman M, Strzelecka T, Dorner L, Schildkraut I, Aggarwal AK. Structure of BamHI endonuclease bound to DNA: partial folding and unfolding on DNA binding. Science. 1995; 269:656–63. PMID: 7624794
24. Gupta YK, Chan SH, Xu SY, Aggarwal AK. Structural basis of asymmetric DNA methylation and ATP-triggered long-range diffusion by EcoP15I. Nat Commun. 2015; 6:7363. Epub 2015/06/13. ncomms8363 [pii] doi: 10.1038/ncomms8363 PMID: 26067164.
25. Wah DA, Hirsch JA, Dorner LF, Schildkraut I, Aggarwal AK. Structure of the multimodular endonuclease FokI bound to DNA. Nature. 1997; 388:97–100. PMID: 9214510
26. Nakonieczna J, Kaczorowski T, Obarska-Kosinska A, Bujnicki JM. Functional analysis of MmeI from methanol utilizer Methylophilus methylotrophus, a subtype IIC restriction-modification enzyme related to type I enzymes. Appl Environ Microbiol. 2009; 75(1):212–23. Epub 2008/11/11. AEM.01322-08 [pii] doi: 10.1128/AEM.01322-08 PMID: 18997032; PubMed Central PMCID: PMC2612229.
27. Vanamee ES, Viadiu H, Kucera R, Dorner L, Picone S, Schildkraut I, et al. A view of consecutive binding events from structures of tetrameric endonuclease SfiI bound to DNA. Embo J. 2005; 24(23):4198–208. PMID: 16308566.
28. Horton JR, Nugent RL, Li A, Mabuchi MY, Fomenkov A, Cohen-Karni D, et al. Structure and mutagenesis of the DNA modification-dependent restriction endonuclease AspBHI. Sci Rep. 2014; 4:4246. Epub 2014/03/08. srep04246 [pii] doi: 10.1038/srep04246 PMID: 24604015; PubMed Central PMCID: PMC3946040.
29. Arnould S, Chames P, Perez C, Lacroix E, Duclert A, Epinat JC, et al. Engineering of large numbers of highly specific homing endonucleases that induce recombination on novel DNA targets. J Mol Biol. 2006; 355(3):443–58. Epub 2005/11/29. S0022-2836(05)01325-2 [pii] doi: 10.1016/j.jmb.2005.10.065 PMID: 16310802.
30. Ashworth J, Havranek JJ, Duarte CM, Sussman D, Monnat RJ Jr., Stoddard BL, et al. Computational redesign of endonuclease DNA binding and cleavage specificity. Nature. 2006; 441(7093):656–9. Epub 2006/06/02. nature04818 [pii] doi: 10.1038/nature04818 PMID: 16738662; PubMed Central PMCID: PMC2999987.
31. Boch J, Scholze H, Schornack S, Landgraf A, Hahn S, Kay S, et al. Breaking the code of DNA binding specificity of TAL-type III effectors. Science. 2009; 326(5959):1509–12. Epub 2009/11/26. 1178811 [pii] doi: 10.1126/science.1178811 PMID: 19933107.
32. Deng D, Yan C, Pan X, Mahfouz M, Wang J, Zhu JK, et al. Structural basis for sequence-specific recognition of DNA by TAL effectors. Science. 2012; 335(6069):720–3. Epub 2012/01/10. science.1215670 [pii] doi: 10.1126/science.1215670 PMID: 22223738; PubMed Central PMCID: PMC3586824.
33. Kim Y-G, Cha J, Chandrasegaran S. Hybrid restriction enzymes: zinc finger fusions to FokI cleavage domain. Proc Natl Acad Sci. 1996; 93:1156–60. PMID: 8577732
34. Mak AN, Bradley P, Cernadas RA, Bogdanove AJ, Stoddard BL. The crystal structure of TAL effector PthXo1 bound to its DNA target. Science. 2012; 335(6069):716–9. Epub 2012/01/10. science.1216211 [pii] doi: 10.1126/science.1216211 PMID: 22223736; PubMed Central PMCID: PMC3427646.

35. Moscou MJ, Bogdanove AJ. A simple cipher governs DNA recognition by TAL effectors. Science. 2009; 326(5959):1501. Epub 2009/11/26. 1178817 [pii] doi: 10.1126/science.1178817 PMID: 19933106.

36. Urnov FD, Rebar EJ, Holmes MC, Zhang HS, Gregory PD. Genome editing with engineered zinc finger nucleases. Nat Rev Genet. 2010; 11(9):636–46. Epub 2010/08/19. nrg2842 [pii] doi: 10.1038/nrg2842 PMID: 20717154.

37. Callahan SJ, Morgan RD, Jain R, Townson SA, Wilson GG, Roberts RJ, et al. Crystallization and preliminary crystallographic analysis of the type IIL restriction enzyme MmeI in complex with DNA. Acta Crystallogr Sect F Struct Biol Cryst Commun. 2011; 67(Pt 10):1262–5. Epub 2011/11/22. S1744309111028041 [pii] doi: 10.1107/S1744309111028041 PMID: 22102043; PubMed Central PMCID: PMC3212378.

38. Otwinowski Z, Minor W. Processing of X-ray diffraction data collected in oscillation mode. Methods Enzymol. 1997; 276:307–26.

39. Fortelle dL, Bricogne G. Maximum-likelihood heavy atom parameter refinement for multiple isomorphous replacement and multiwavelength anomalous diffraction methods. Methods Enzymol. 1997; 276:472–94.

40. Emsley P, Cowtan K. Coot: model-building tools for molecular graphics. Acta Crystallogr D Biol Crystallogr. 2004; 60(Pt 12 Pt 1):2126–32. PMID: 15572765.

41. Adams PD, Afonine PV, Bunkoczi G, Chen VB, Davis IW, Echols N, et al. PHENIX: a comprehensive Python-based system for macromolecular structure solution. Acta Crystallogr D Biol Crystallogr. 2010; 66(Pt 2):213–21. Epub 2010/02/04. S0907444909052925 [pii] doi: 10.1107/S0907444909052925 PMID: 20124702; PubMed Central PMCID: PMC2815670.
